# Subretinal Coapplication of Tissue Plasminogen Activator and Bevacizumab with Concurrent Pneumatic Displacement for Submacular Hemorrhages Secondary to Neovascular Age-Related Macular Degeneration

**DOI:** 10.4274/tjo.galenos.2020.72540

**Published:** 2021-02-25

**Authors:** Remzi Avcı, Ayşegül Mavi Yıldız, Esat Çınar, Sami Yılmaz, Cem Küçükerdönmez, Fatma Duriye Akalp, Emre Avcı

**Affiliations:** 1 Bursa Retina Eye Hospital, Bursa, Turkey; 2 Ekol Eye Hospital, İzmir, Turkey; 3 Acıbadem University Faculty of Medicine, İstanbul, Turkey

**Keywords:** Age-related macular degeneration, anti-VEGF, submacular hemorrhage, tissue-plasminogen activator

## Abstract

**Objectives:**

To evaluate the functional and morphological outcomes of vitrectomy in combination with intravitreal 5% C3F8 tamponade and subretinal injections of tissue plasminogen activator (tPA) and anti-vascular endothelial growth factor (anti-VEGF) in patients with submacular hemorrhage (SMH) and to investigate the preoperative prognostic factors.

**Materials and Methods:**

This retrospective study included 30 patients (16 women, 14 men) diagnosed with SMH secondary to neovascular age-related macular degeneration (AMD). Preoperative SMH thickness and area, ellipsoid zone integrity, and postoperative reduction in the amount of subfoveal blood on optical coherence tomography and fundus photographs were assessed. Furthermore, visual acuity (VA), hemorrhage duration, and the need for additional intravitreal anti-VEGF injections were recorded.

**Results:**

The patients’ mean age was 73.33±8.23 years. Mean VA improved from logMAR 2.11±0.84 at baseline to logMAR 1.32±0.91, 0.94±0.66, 1.13±0.84, and 1.00±0.70 at postoperative month 1, 2, 3, and 6, respectively. A significant negative correlation was found between hemorrhage duration and postoperative VA at month 2 (p=0.005), month 3 (p=0.019), and month 6 (p=0.012). The mean preoperative SMH duration was significantly shorter in patients who achieved total resolution of the hemorrhage compared with the subtotal resolution group (p<0.001). The mean SMH area was smaller in the patients with continuous ellipsoid zone.

**Conclusion:**

Vitrectomy and submacular tPA and anti-VEGF injections with concurrent C3F8 tamponade appears to provide adequate displacement of the hemorrhage, resulting in significant VA improvement in patients with hemorrhagic neovascular AMD. Timing of the surgery appears to be the most important factor determining the final VA.

## Introduction

Submacular hemorrhage (SMH) is a clinical condition that frequently originates from age-related macular degeneration (AMD), polypoidal choroidal vasculopathy (PCV), macro-aneurysm, trauma, myopic choroidal neovascularization, and intraocular tumors, and may lead to significant loss of vision in case of a delay in treatment.^1,2^ Hemorrhage in the submacular area may cause irreversible destruction of the retinal pigment epithelium (RPE) and photoreceptors as early as 24 hours after onset, as a result of iron-based toxicity caused by reactive oxygen radicals. Furthermore, fibrin-based tractional forces and the physical barrier effect of the hemorrhage obstructing the visual axis are other well-known causes of the visual impairment.^3^

Therefore, prompt removal of the hemorrhage is critical to prevent irreversible vision loss. Current treatment strategies for SMH include pneumatic displacement of the hemorrhage from the macula, pharmacologic fibrinolysis achieved with tissue plasminogen activator (tPA), and application of anti-vascular endothelial growth factor (anti-VEGF) agents in case of underlying wet AMD or PCV.^4,5,6,7,8,9,10^ These techniques have been performed alone or in combination with vitrectomy.

The primary objective of this study was to determine the effectiveness of a standardized surgery including vitrectomy, submacular injections of tPA and anti-VEGF with a concurrent C_3_F_8_ tamponade to displace the blood in patients with SMH secondary to neovascular AMD. The secondary objective was to assess the preoperative prognostic factors associated with the final visual outcome and postoperative degree of SMH resolution.

## Materials and Methods

This retrospective study included 30 patients (16 females, 14 males) diagnosed with SMH secondary to neovascular AMD between January 2014 and March 2018. Pars plana vitrectomy in combination with intravitreal injection of 5% C_3_F_8_ gas, subretinal injection of tPA (Actilyse, 10 mg/mL, Boehringer-Ingelheim, Germany) and anti-VEGF (bevacizumab 1.25 mg/0.05 mL) was performed for all the participants. Each patient was informed about the risks and benefits of the surgery and written informed consent was obtained. All procedures performed in this study were in accordance with the ethical standards of the institutional and national research committee and with the 1964 Helsinki Declaration and its later amendments or comparable ethical standards.

A complete ophthalmic examination including assessment of best-corrected visual acuity (BCVA) on the Snellen chart, biomicroscopic anterior and posterior segment examination, measurement of intraocular pressure via applanation tonometry, spectral-domain optical coherence tomography (SD-OCT) (Spectralis; Heidelberg Engineering, Heidelberg, Germany), color fundus photography, fluorescein angiography (FA), and indocyanine green angiography (ICGA) (HRA-2; Heidelberg Engineering, Heidelberg, Germany) were performed preoperatively if needed. A radial line scan pattern which uses 24 radial B-scans centered on the fovea was used for SD-OCT imaging. The caliper feature of the SD-OCT was used to measure the thickness of the subfoveal hemorrhage and the thickest scan was included. Color fundus photographs were obtained through dilated pupils using Topcon TRC-NW8F digital imaging device (Topcon Medical Systems, Inc., NJ, USA) and the area of the SMH was calculated in square millimeters using the manual caliper function of the ImageNet software using the method described by Shin et al.^11^

Additionally, the integrity of the subfoveal ellipsoid zone (EZ) was assessed in the horizontal and vertical SD-OCT scans and classified as continuous if it was clearly seen without any interruption in both scans and as discontinuous if it appeared blurred or interrupted in the vertical or horizontal images. All measurements were performed by the same researcher to ensure consistency.

### Surgical Technique

Surgical procedures were performed by the same experienced vitreoretinal surgeon (R.A.). Standard 3-port vitrectomy was performed for all the patients using 23-gauge (G) vitrectomy system (DORC, Dutch Ophthalmic Research Center, Zuidland, the Netherlands) and a non-contact viewing system (EIBOS 2, Carl Zeiss Meditec, Jena, Germany). Posterior vitreous detachment was induced if not present after core vitrectomy. Injections of tPA (25 µg/0.1 mL, range: 0.1-0.2 mL) and anti-VEGF (bevacizumab 1.25 mg/0.05 mL) were performed consecutively through separate needles (DORC extendible 41G subretinal injection needle, 23G/0.6 mm). The retina was penetrated inferotemporally, approximately 3 disc diameters from the fovea in the absence of significant sub-RPE hemorrhage. However, in case of a prominent sub-RPE component confirmed on preoperative OCT, an injection site far from the RPE detachment area was selected to avoid penetration under the RPE. Moreover, subretinal injection was performed at two separate sites in 8 of the patients with widespread SMH.

Intravitreal gas (C_3_F_8_, 5%) tamponade was then administered to achieve 75% filling of the vitreous cavity postoperatively. Following initial prone positioning, patients were instructed to spend most of the day in reading position (head at a 45º angle to the ground) for 5 days postoperatively.

The patients were called for follow-up examinations at postoperative week 1, month 1, month 2, month 3, and month 6. In addition to the routine ophthalmic examination, fundus photography and SD-OCT assessment, FA, and ICGA were repeated if necessary at the follow-up visits. Patients who presented with active choroidal neovascular membrane were treated with additional anti-VEGF injections.

Displacement of the SMH was categorized as total or subtotal at the final visit. Total resolution was defined as clearance of the entire subretinal hemorrhage, whereas subtotal resolution was defined as a mean subfoveal hemorrhage thickness of less than 100 µm within a 1,500-µm radius of the foveal center on horizontal and/or vertical SD-OCT scans.^12^ The preoperative duration of the SMH was determined according to symptom onset.

The main outcome measure of this study was to evaluate the prognostic factors affecting final visual outcome for patients with SMH secondary to neovascular AMD. Secondary outcome measures included visual outcomes and blood displacement at final follow-up, and tertiary outcome measures were complication and recurrence rates.

### Statistical Analysis

Statistical analyses were performed using SPSS software (version 21.0 for Windows; IBM Corp, Armonk, NY, USA). For each independent variable, conformity to normal distribution was tested with the Kolmogorov-Smirnov test. Preoperative and postoperative BCVA were evaluated using the paired samples t-test. Independent groups were evaluated using the independent samples t-test. Correlations between preoperative SMH features (duration, thickness, and area) and postoperative visual acuity were assessed using Pearson correlation test. Categorical variables were compared between groups using Fisher’s exact test. A p-value less than 0.05 was accepted as statistically significant.

## Results

**Figure 1:**
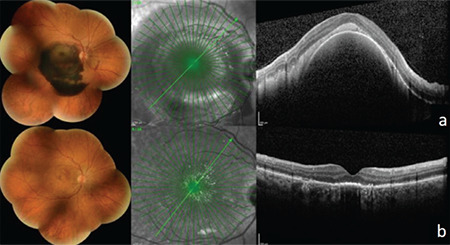
Representative images of a patient diagnosed with an extensive submacular hemorrhage reaching beyond the major vascular arcades. Ultrawidefield color fundus photograph and B scan SD-OCT images at presentation (a) and postoperative month 2 (b) SD-OCT: Spectral domain-optical coherence tomography

**Figure 2:**
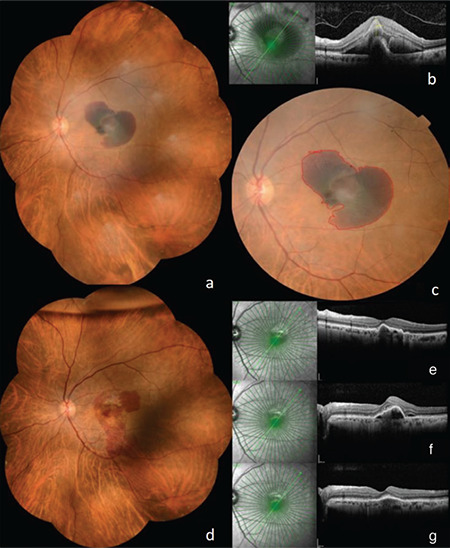
Representative images of a patient diagnosed with a localized submacular hemorrhage involving the fovea. Ultra-widefield color fundus photography (a) and B scan SD-OCT image (b) at presentation. Color fundus photograph of the same patient demonstrating the area measurement of the hemorrhage (c). Ultra-widefield color fundus photography at postoperative month 1; gas tamponade is visible at the top of the image (d). B scan SD-OCT image at month 1 (e), month 2 (f), and month 6 (g) SD-OCT: Spectral domain-optical coherence tomography

Regarding the demographics of the study group, 16 patients were women, 14 were men, and the mean (± standard deviation [SD]) age was 73.33±8.23 years. All patients’ preoperative diagnosis was neovascular AMD (n=30). The mean duration of the hemorrhage prior to surgery was 13.70±8.05 (range: 2-30) days. The mean thickness and area of the hemorrhage at presentation were 738.91±280.88 (range: 415-1476) µm and 61.95±43.47 (range: 10.75-176.42) mm^2^, respectively. Total (n=16, 53.3%) or subtotal (n=14, 46.7%) resolution of the hemorrhage was achieved in all patients. There were no complications perioperatively in any of the patients. Two representative cases showing gradual resolution of the hemorrhage on both SD-OCT and color fundus photographs are shown in Figure 1 and Figure 2.

**Figure 3:**
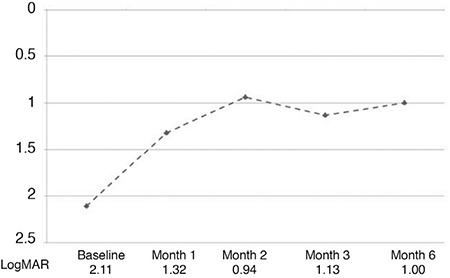
Changes in BCVA in LogMAR units during the study period BCVA: Best-corrected visual acuity

Mean BCVA showed a gradual improvement from preoperative logMAR 2.11±0.84 (Snellen equivalent 20/2576) to logMAR 1.32±0.91 (Snellen equivalent 20/458), logMAR 0.94±0.66 (Snellen equivalent 20/174), logMAR 1.13±0.84 (Snellen equivalent 20/269), and logMAR 1.00±0.70 (Snellen equivalent 20/200) at postoperative month 1, 2, 3, and 6, respectively. The improvement in BCVA was significant at all follow-up visits compared with baseline (p<0.05) (Figure 3). Neither age nor sex was found to be correlated with final VA. Similarly, no significant correlation was detected between preoperative mean BCVA, hemorrhage thickness and area, and postoperative VA at month 1, 2, 3, and 6 (p>0.05 for all).

A significant negative correlation was found between preoperative hemorrhage duration and postoperative VA at month 2 (r=0.50, p=0.005), month 3 (r=0.44, p=0.019), and month 6 (r=0.53, p=0.012). The patients were divided according to preoperative duration of hemorrhage into two groups, the early treatment group (duration <10 days; n=10, 33.3%) and the delayed treatment group (duration ≥10 days; n=20, 66.7%) (Table 1). A significantly greater improvement in BCVA was attained in the early treatment group at postoperative visits compared with the delayed treatment group (p=0.014, p=0.006, and p=0.004 respectively for postoperative month 2, 3, and 6).

Mean preoperative SMH duration was significantly shorter in patients who achieved total displacement of the hemorrhage (n=16, average 9 days) compared with patients who achieved subtotal displacement (n=14, average 19 days) (p<0.001) (Table 2). Furthermore, mean BCVA was statistically significantly higher at postoperative month 2, 3, and 6 in the total resolution group (p<0.05 for all). Moreover, the patients were divided into two groups according to ellipsoid zone status on preoperative SD-OCT: disrupted (n=23, 76.7%) or continuous (n=7, 23.3%) (Table 3). At postoperative month 1, 2, 3, and 6, no statistically significant difference was observed in terms of BCVA levels between two groups (p>0.05). The area of the macular scar, which was measured for 22 of the patients, showed no statistically significant correlation with mean preoperative SMH duration (p=0.768). No statistically significant difference was detected between the early treatment and delayed treatment groups in terms of scar size at final visit (p=0.548). During follow-up visits, 29 additional anti-VEGF injections were required for 12 of the eyes (ranibizumab, Novartis Pharma AG, Basel, Switzerland, for 10 eyes; aflibercept, Eylea®, Regeneron Pharmaceuticals, Inc., Tarrytown, NY, USA for 2 eyes) (Table 4). The baseline SMH area was significantly smaller in the additional anti-VEGF treatment group (p=0.003). At postoperative month 1, rehemorrhage without foveal involvement was detected in 2 of the eyes, which was managed with additional anti-VEGF injections.

## Discussion

In the current study, we investigated the prognostic factors affecting the final visual outcome in patients with SMH secondary to neovascular AMD. Furthermore, we evaluated the anatomical and functional results of this standardized surgery. Total or subtotal displacement of the hemorrhage was achieved in all of the patients at final follow-up. The results demonstrated that mean BCVA improvement was statistically significant at all follow-up visits. The duration of the SMH seemed to be the most important prognostic factor influencing final visual acuity. Moreover, patients with shorter disease duration had a significantly higher rate of total hemorrhage resolution. Patients with mean SMH duration less than 10 days had a significantly higher probability of achieving total resolution of the hemorrhage compared to eyes with SMH duration of 10 days or longer. However, the area and thickness measurements of the hemorrhage were not a significant predictor of outcome.

There remains no consensus on optimal treatment of SMH, as coexisting macular pathologies and duration of SMH may influence the choice of treatment and outcomes.^12,13,14^ Techniques including vitrectomy and direct subretinal removal of the SMH have been found ineffective. In last couple of years, intravitreal/subretinal injection of tPA with a concurrent nonexpansile gas tamponade have become preferred methods.^9,15,16^ Pars plana vitrectomy has been reserved for large SMHs, whereas intravitreal injection of tPA/anti-VEGF is preferred for localized hemorrhages. However, vitrectomy in combination with subretinal tPA, anti-VEGF and pneumatic displacement appears to be more favorable than intravitreal applications alone. It has also been suggested that diffusion of the tPA through the retina is limited because of its high molecular weight when applied intravitreally.^17^

In the current study, a significant BCVA improvement was observed at postoperative visits in all patients. In addition, visual acuity gain remained stable during the follow-up period. The greatest response in terms of improvement of the mean BCVA was achieved at postoperative month 2. Similarly, Chang et al.^15^ presented a study which they performed pars plana vitrectomy, subretinal tPA injection, and intraocular gas tamponade with or without injection of anti-VEGF for thick SMH due to AMD (n=101). They reported that the visual acuity improved by at least 1 line in 82% and 3 lines or more in 19.6% of the eyes. Moreover, patients who received postoperative regular anti-VEGF injection showed greater improvement in BCVA at postoperative month 6 compared with the group who did not receive additional anti-VEGF. Nevertheless, no significant relationship was detected between preoperative hemorrhage duration and final BCVA in that study.

Arias et al.^18^ performed early (within the first 5 days) injection of subretinal tPA for 8 eyes with thick SMH due to AMD and reported a significant improvement in BCVA as well as total resolution of the hemorrhage in all eyes.

Of the eyes in the current study, total SMH resolution was observed in 16 (53.3%) whereas subtotal resolution was observed in 14 (46.6%). Furthermore, mean preoperative hemorrhage duration was significantly shorter in the patients who showed total displacement of the hemorrhage compared to those with subtotal resolution postoperatively. In earlier studies, it was reported that delayed surgery may cause organization of the hemorrhage and lower the probability of achieving liquefaction with tPA, which may result in inadequate peripheral displacement.^19,20^ It has also been reported in the literature that the clinical results obtained in subretinal hemorrhages with a duration more than 2 weeks are associated with poor outcomes and this supports the results of the current study.^21^

In a recent study, Hirashima et al.^22^ analyzed preoperative prognostic factors of visual function. They performed PPV in combination with subretinal tPA injection for 9 eyes with SMH due to AMD. They reported that shorter duration before surgery and hemorrhage thickness less than 400 µm were associated with more favorable functional outcomes. Moreover, the researchers evaluated the preoperative OCT images in regard to EZ integrity and they reported better final visual outcomes in patients in eyes with an intact EZ layer. However, no relationship was found between preoperative hemorrhage area and postoperative BCVA. In our study, patients who received treatment earlier than 10 days showed better visual acuity at all postoperative follow-up visits. These results support the researchers suggesting that iron-based toxicity to photoreceptors and RPE is time dependent.^9,23^ Schulze et al.^21^ measured preoperative hemorrhage thickness with B-scan ultrasonography and observed a negative correlation between hemorrhage thickness and final visual outcome in univariate statistical analyses, although final visual acuity was not significantly associated with age and hemorrhage duration or thickness in multiple regression analyses. The researchers stated that the area of the hemorrhage and preoperative visual acuity were the significant prognostic factors influencing functional outcomes. Hirashima et al.^22^ reported no linear correlation between the hemorrhage thickness and postoperative visual acuity, except for the eyes with a SMH thicker than 400 micron, who showed significantly poorer functional results. However, we did not detect a significant relationship between final BCVA and preoperative thickness, area measurements, or EZ continuity.

In a study reported by Fassbender et al.^24^, three different approaches for SMH secondary to AMD were evaluated. An interesting finding was that the final scar sizes in eyes that underwent vitrectomy with subretinal tPA injection were much smaller than in those that received intravitreal gas alone (pneumatic displacement) and those that received a combination of intravitreal gas and tPA. Previous studies in the literature reported that very early surgery might increase the risk of rehemorrhage; however, delayed interventions exceeding the critical time interval might result in organization of the hemorrhage which becomes resistant to liquefaction and displacement.^19,20^ In respect to critical surgical timing, Kumar et al.^9^ reported a mean time of 23 days, whereas Meyer et al.^25^ reported 9 days. In the current study, this time span was found to be 10 days.

### Study Limitations

Despite obtaining good clinical and morphological results, the current study had some limitations such as retrospective design, a relatively limited number of patients, and short follow-up time. However, a single treatment protocol was performed by a single experienced vitreoretinal surgeon for all patients, which allows investigation of the effects of other variables on prognosis.

## Conclusion

According to the results of this study, surgical timing appears to be the most important prognostic factor influencing final visual acuity. As SMH can cause irreversible photoreceptor damage, the probability of achieving a favorable outcome is lower among patients with hemorrhage duration of less than 10 days. In addition, early surgical intervention appears to be associated with higher rates of total displacement of the hemorrhage. Further studies are needed to analyze the prognostic factors affecting functional and anatomical outcomes.

## Figures and Tables

**Table 1:**
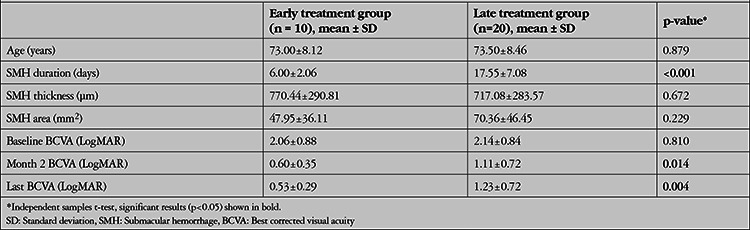
General characteristics of two groups in which early (<10 days after symptom onset) or delayed (>10 days) surgical intervention was performed

**Table 2:**
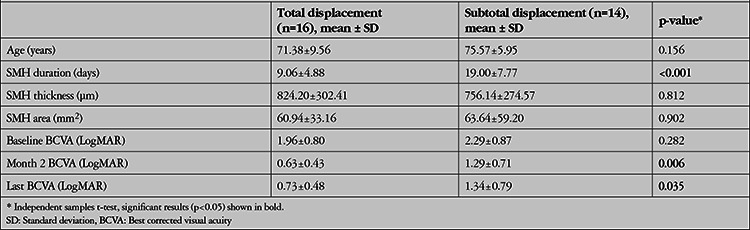
General characteristics of two groups in which total or subtotal resolution of the submacular hemorrhage (SMH) was achieved

**Table 3:**
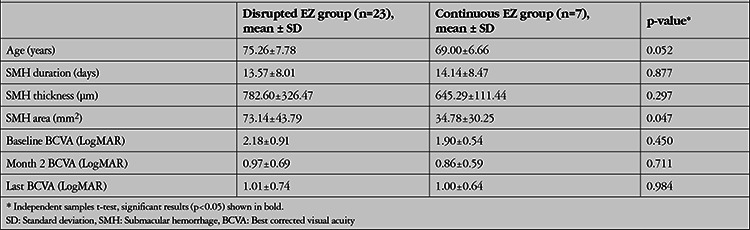
General characteristics of two groups based on the preoperative status of the ellipsoid zone (EZ)

**Table 4:**
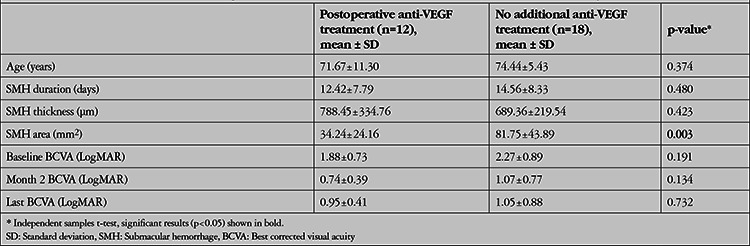
General characteristics of two groups based on postoperative need for additional anti-VEGF injections
